# Facile one-pot construction of copper-loaded polydopamine films on NiTi alloy for adjustable antibacterial activity and enhanced corrosion resistance

**DOI:** 10.1039/d5ra08002a

**Published:** 2026-01-21

**Authors:** Ying Li, Hongfeng Liu, Tong Liu, Hao Liu, Leheng Ren, Haojie Sun, Yongkui Yin

**Affiliations:** a College of Life Science, Mudanjiang Medical University Mudanjiang 157011 Heilongjiang Province PR China yinyongkui@mdjmu.edu.cn; b School of Basic Medicine, Mudanjiang Medical University Mudanjiang 157011 Heilongjiang Province PR China; c School of Health Management, Mudanjiang Medical University Mudanjiang 157011 Heilongjiang Province PR China

## Abstract

Nickel–titanium (NiTi) alloys are widely employed as implantable devices, but their intrinsic poor antibacterial activity and Ni-ion release remain a major challenge. Leveraging copper's antibacterial efficacy, we developed a facile one-pot route to construct copper-loaded polydopamine films on NiTi alloy (Cu@PDA/NiTi). In a dopamine–CuSO_4_ mixture, dopamine simultaneously polymerizes and reduces Cu^2+^, the resulting Cu^2+^ coordinates with PDA to form a metal-phenolic network (MPN), which incorporates both Cu^2+^ and Cu nanoparticles (NPs). By tuning CuSO_4_ concentration (*C*_CuSO_4__) from 1 to 30 mM, the Cu@PDA/NiTi possesses adjustable Cu content and antibacterial potency. Antibacterial ratios toward *Escherichia coli* (*E. coli*) and *Staphylococcus aureus* (*S. aureus*) reach 46.3–99.4% and 47.5–97.3%, respectively, while Ni-ion release is markedly suppressed and corrosion resistance significantly enhanced. *In vitro* and *in vivo* assays confirm excellent biosafety. This scalable strategy offers a practical avenue for customizable antibacterial NiTi implants and inspires the design of next-generation functional biomaterials.

## Abstract 2

A facile one-pot construction of Cu@PDA film on NiTi alloy imparts adjustable antibacterial activity, enhanced corrosion resistance, and inhibited Ni-ion release, addressing key limitations for implant applications.

## Graphical abstract

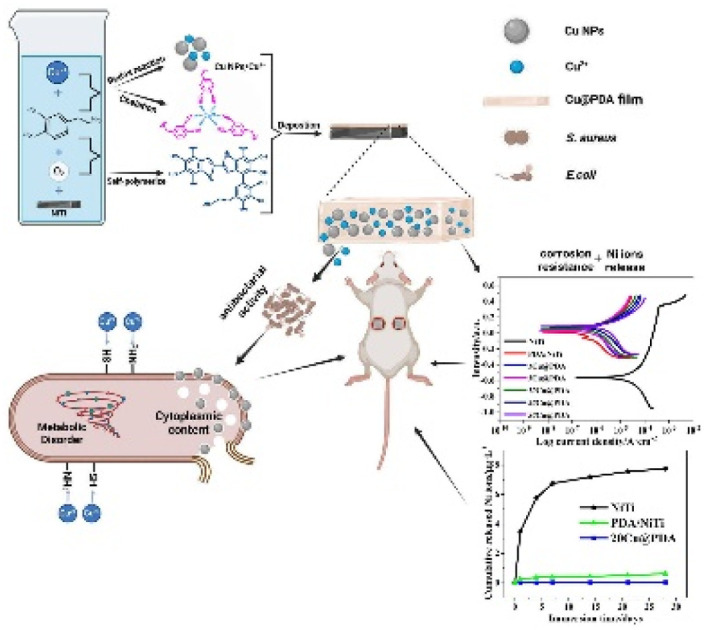

## 1. Introduction

Advances in medical technology, population growth, and rising rates of trauma have driven unprecedented demand for biomaterials.^1,2^ The rapid growth of biomaterial development has led to an increase in metallic biomaterials.^3^ Among metallic implants, NiTi alloy stands out because of its unique mechanical properties,^4,5^ most notably the shape memory effect and superelasticity,^6^ enabling its widespread use in orthopaedics,^7^ dentistry,^8^ cardiovascular stents,^9^ orthodontics and surgical instruments.^10^ However, the intrinsic lack of antibacterial activity and sustained release of Ni ions from NiTi alloy markedly increase the risk of post-surgical infections and raise concerns regarding carcinogenicity and teratogenicity, thereby severely restricting their clinical utility.^11–13^ Surface engineering to simultaneously enhance antibacterial activity and corrosion resistance is therefore imperative for the safe and effective deployment of NiTi implants.^14,15^

Implant-associated infections remain a formidable clinical challenge.^16,17^ Traditional therapeutic approaches, such as systemic antibiotic therapy suffer from sub-therapeutic local concentrations and rapid emerging resistant bacteria, resulting in frequent treatment failures.^18,19^ Inorganic metal antimicrobials-namely silver (Ag), copper (Cu), and zinc (Zn) have therefore attracted increasing interest.^20,21^ Ag nanoparticles (NPs) exemplify this trend: released Ag^+^ can perforate bacterial cell walls, disrupt metabolic processes, and generate reactive oxygen species (ROS), thereby achieving high antibacterial efficacy.^22^ However, the broad application of Ag NPs is constrained by high cost, and complex synthesis, and potential cytotoxicity.^23–25^ By contrast, Cu offers a compelling alternative. Its intrinsic antibacterial efficacy is inferior to that of Ag, while its low cost, natural abundance, essential element in human body,^26^ and promoting wound healing^27^ make it clinically attractive. Cu NPs exhibit bactericidal activity both directly and through the Cu^2+^ ions they release. Together, they disrupt bacterial envelopes, elicit a burst of reactive oxygen species (ROS), yielding a potent and durable antimicrobial effect.^28,29^ Despite these advantages, current strategies for integrating Cu into NiTi surfaces—such as micro-arc oxidation or electrochemical deposition—either fail to enhance corrosion resistance or lack tunability to meet the variable antibacterial demands of diverse implants. Consequently, a facile and scalable surface modification approach that simultaneously delivers controllable Cu release and improved corrosion resistance is urgently required.

Polydopamine (PDA) films, widely employed for surface modification, possess intrinsic redox activity that *in situ* reduces metal ions to nanoparticles, conferring potent antibacterial activity,^30,31^ while the PDA matrix itself markedly enhances the substrate's corrosion resistance.^32,33^

**Scheme 1:**
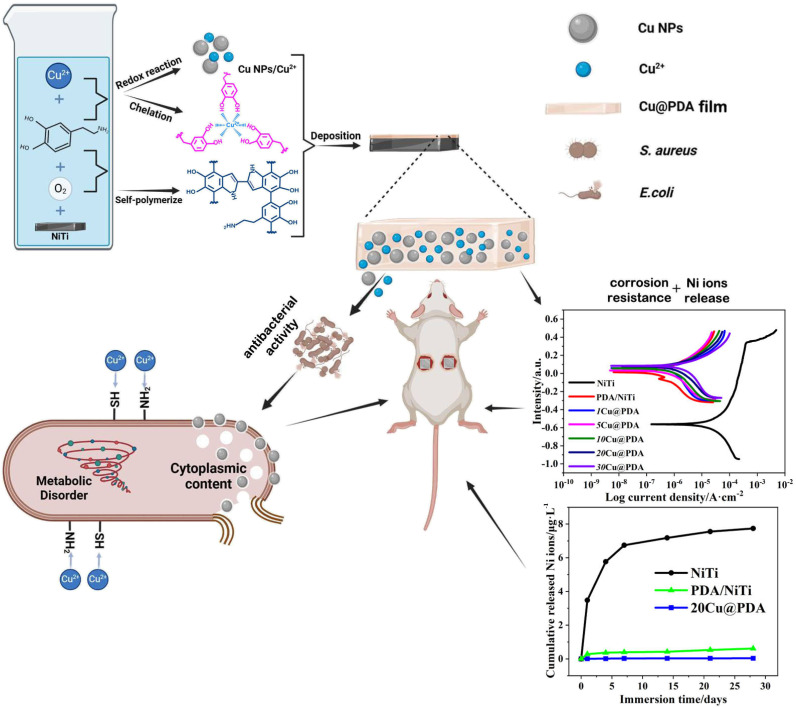
The elaborate design considerations of this study.

Building on our previous one-step Ag@PDA strategy,^34^ we now extend this concept to construct Cu@PDA/NiTi. By immersing NiTi alloys into dopamine–CuSO_4_ mixture, dopamine polymerization and Cu^2+^ reduction proceeded simultaneously, constructing Cu@PDA/NiTi. In this process, Cu^2+^ coordinates chelation with PDA oligomers to form a stable metal-phenolic network (MPN), which effectively incorporates both Cu^2+^ and Cu NPs. Simply adjusting the *C*_CuSO_4__ from 1 mM to 30 mM precisely adjusts the Cu content and thus the antibacterial activity, allowing the Cu@PDA/NiTi to meet the diverse infection-control demands of various implant sites while effectively suppressing Ni-ion release and improving corrosion resistance. The elaborate design considerations of this study are demonstrated in Scheme 1.

This work presents a strategy to address the critical challenges of NiTi alloy by constructing a functional coating. The constructed Cu@PDA/NiTi concurrently tackles the issues of Ni-ion release and corrosion resistance of NiTi alloy, while providing tunable antibacterial activity *via* simple adjustment of the initial *C*_CuSO_4__. The novelty of this study lies in this demonstrated concurrent resolution of both limitations of NiTi alloy through a single one-pot process.

## 2. Experimental section

### 2.1 Materials and preparation

Plates of equiatomic NiTi with dimensions of 10 mm × 10 mm × 2 mm were progressively ground with 600–2000 grit SiC sandpapers and mirror-polished with diamond polishing agent (1.5 µm). Following sequential ultrasonic cleaning in acetone, ethanol and deionized water (10 minutes per solvent), the plates were air-dried, then immersed in 20 mL of a freshly prepared solution containing CuSO_4_ at specified concentrations (1, 5, 10, 20, 30 mM) and dopamine hydrochloride (2 mg mL^−1^, Sigma-Aldrich). The solution pH value was adjusted to 8.5 using Tris base. The reaction proceeded at 25 °C for 12 hours in darkness under gentle shaking (50 rpm, 20 mm amplitude, SHA-BA, Shanghai Guning). After withdrawal, the plates were ultrasonically rinsed in deionized water (5 minutes) and vacuum-dried at 40 °C. The resulting Cu@PDA/NiTi are denoted *m*Cu@PDA, where “*m*” denotes the value of initial concentration of CuSO_4_ (in mM).

### 2.2 Characterizations

Surface morphology and microstructure were investigated using transmission electron microscopy (TEM, JEOL, JEM-2100, Japan) equipped with energy-dispersive X-ray spectroscopy (EDS, Bruker, Xflash600, Germany) mapping. Chemical compositions were analyzed by X-ray photoelectron spectroscopy (XPS, Thermo Fisher Scientific, ESCAL AB2502Xi, USA). Using Al Kα as the excitation source, the test power is set to 300 W, and C 1s (binding energy of 284.6 eV) is used to correct the charge shift of the test element. Fourier transform infrared spectroscopy (FTIR, Thermo Fisher Scientific, NicoletIS10, USA). For TEM, films were carefully peeled off from the substrates, dispersed in ethanol, and dropped onto carbon grids. Observation of morphology and high-resolution analysis using acceleration voltages of 100 kV and 200 kV respectively. Additionally, FTIR pellets were prepared by grinding dried films with KBr. The scanning range is 400–4000 cm^−1^. Surface topography and roughness were assessed by atomic force microscopy (AFM, Bruker, USA) operating in tapping mode.

Hydrophilicity was evaluated *via* the sessile drop method using a contact angle system (OCA, Dataphysics, OCA25, Germany). A 5 µL droplet of deionized water was gently placed on each sample, and the static contact angle was measured.

### 2.3 Corrosion resistance and ion release kinetics

Electrochemical corrosion behavior was assessed in Hank's solution (pH = 7.4) at 37 °C using a standard three-electrode setup on an electrochemical workstation (Autolab, Metrohm AG, PGSTAT204, Switzerland). The system comprised a saturated calomel electrode (SCE), a platinum foil, and the specimen with 1 cm^2^ exposed area acted as the working electrode. After immersing the samples in Hank's solution to stabilize the open-circuit potential (OCP) for 7200 seconds, potentiodynamic polarization scans were conducted starting from 400 mV below the OCP and scanning anodically to approximately 500 mV *vs.* SCE at a rate of 0.5 mV s^−1^.

The release profiles of Cu^2+^ and Ni ions were monitored over 1, 4, 7, 14, 21, and 28 days using inductively coupled plasma optical emission spectrometry (ICP-OES, Leeman, Prodigy High Dispersion, USA). The test samples were placed in 5 mL of PBS and maintained at 37 °C under dark conditions. The PBS was replaced at each time point.

### 2.4 *In vitro* antibacterial evaluation

Antibacterial activity was evaluated *in vitro* against *E. coli* and *S. aureus* (10^6^ CFU mL^−1^) using a modified plate counting technique. A 50 µL aliquot bacterial suspension was gently dropped onto the sample surface (1 cm^2^), covered with a sterilized plastic film, and incubated at 37 °C in darkness under humid conditions for 24 hours. After incubation, the sample, bacterial suspension, and plastic film were collected into a sterile 10 mL centrifuge tube containing 5 mL of PBS, followed by vortexing for 5 minutes. The resulting suspensions were serially diluted, plated on LB agar, and incubated at 37 °C for 18 hours before colony counting. The antibacterial ratio (AR) was calculated based on eqn (1):^35,36^

1 AR = [(CFU_control_ − CFU_sample_)/CFU_control_] × 100%

where CFU_control_ and CFU_sample_ represent the average CFU counts of the control group and sample treated group, respectively.

### 2.5 *In vitro* cytotoxicity assessment

*In vitro* cytotoxicity was evaluated using mouse embryonic fibroblast NIH-3T3 cells. Cells were maintained in DMEM supplemented with 10% fetal bovine serum and 1% penicillin–streptomycin at 37 °C and 5% CO_2_. A suspension (5 × 10^4^ cells mL^−1^) was seeded in 24-well plates containing the test samples. Following co-cultivation for 1, 3, and 5 days, the CCK-8 assay was performed by adding 50 µL of the reagent to each well and incubating for 4 hours. Subsequently, aliquots of 100 µL were then transferred to a 96-well plate for measurement. In this assay, DMEM alone was designated as the negative control, while DMEM containing 10% DMSO served as the positive control. Absorbance was read at 570 nm (reference 630 nm) using a microplate reader (Molecular Devices, M3). All measurements were performed in triplicate.

Additionally, cell viability and morphology were assessed by fluorescence microscopy. Briefly, Calcein-AM and propidium iodide (PI) were co-diluted in PBS and shielded from light. After aspirating the culture medium, cells were gently rinsed three times with PBS, incubated with 1 mL of the AM/PI mixture for 15 minutes at 37 °C in the dark, and then imaged on an inverted fluorescence microscope (Leica DMI8). Quantitative viability data were acquired with ImageJ software.

### 2.6 Subcutaneous implantation and biological security evaluation

Male ICR mice (25–30 g, 6–8 weeks) were supplied by Beijing Vital River Laboratory Animal Technology Co., Ltd. All procedures were approved by the Institutional Animal Care and Use Committee of Mudanjiang Medical University (IACUC-20240910-305). Twenty mice were randomly allocated into four groups (*n* = 5 per group): polished NiTi, PDA/NiTi, Cu@PDA/NiTi, and control groups. Under sterile conditions, two 1 cm longitudinal incisions were made on the bilateral dorsal surfaces. Corresponding samples (5 cm × 5 cm × 5 cm) were implanted into the incisions, and the skin was closed with sutures. After 28 days, the mice were euthanized, and major organs (heart, liver, spleen, lung, and kidney) were harvested and fixed in 4% paraformaldehyde. Hematoxylin and Eosin (H&E) staining was then performed to evaluate any significant pathological changes or organ damage.

### 2.7 Statistical analysis

The data and images were processed using Origin Pro 2021 and GraphPad Prism 8 software. All performance data derived from the samples were measured in triplicate, and one-way ANOVA was applied for comparison between multiple groups, with *P* < 0.05 considered statistically significant.

## 3. Results and discussions

### 3.1 Composition and surface morphology

**Fig. 1:**
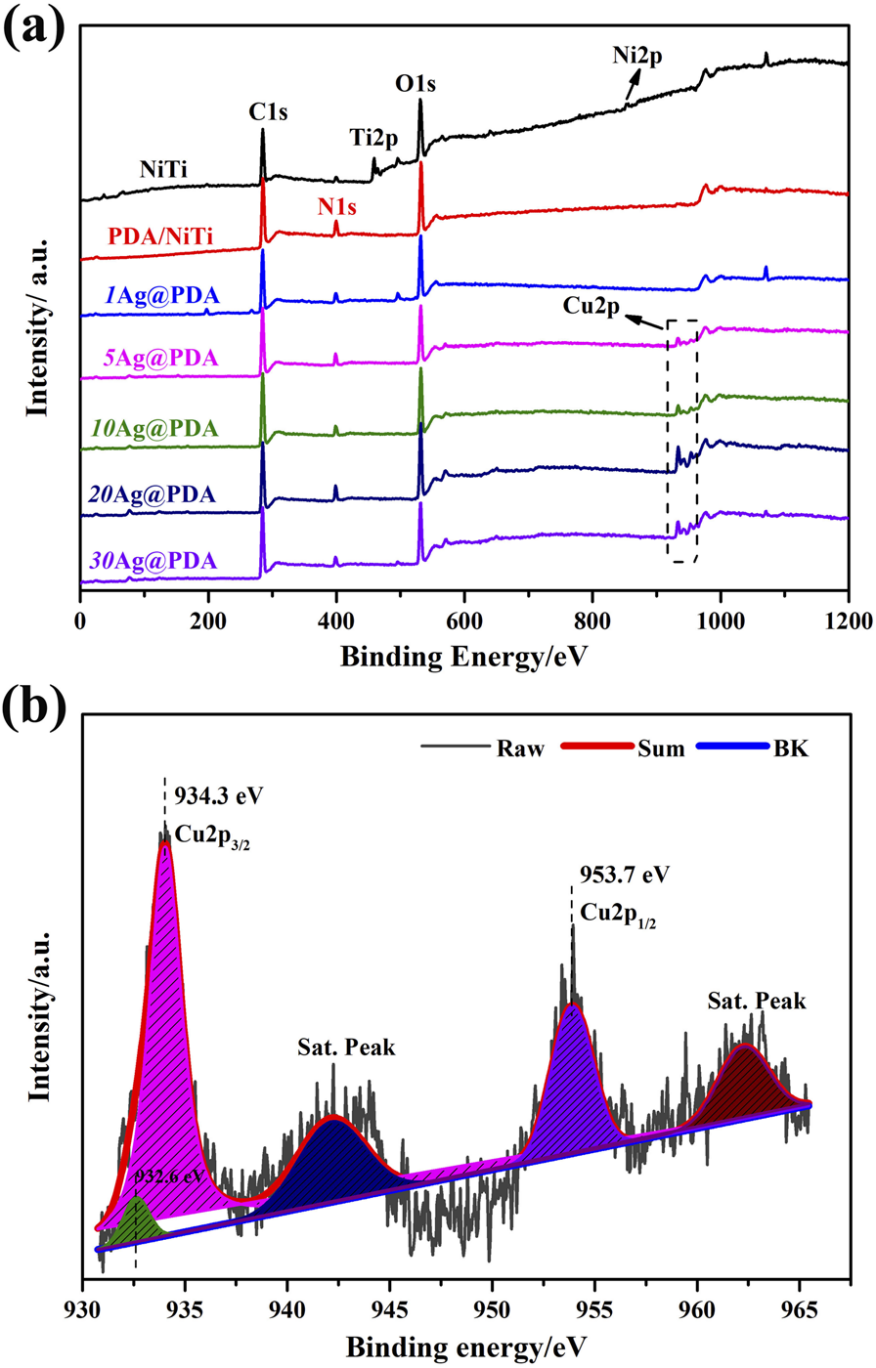
XPS spectra of polished NiTi, PDA/NiTi and Cu@PDA/NiTi constructed by adjusting *C*_CuSO_4__, (a) full spectra and (b) high-resolution spectra of 20Cu@PDA.

Fig. 1 presents the XPS spectra of polished NiTi, PDA/NiTi and Cu@PDA/NiTi constructed by adjusting *C*_CuSO_4__, with the corresponding elemental compositions listed in Table S1. As shown in Fig. 1a, the NiTi spectrum exhibits characteristic Ti 2p (480 eV) and Ni 2p (850 eV) peaks, while minor C 1s (280 eV) and N 1s (400 eV) signals arise from air contamination. After PDA film deposition, the Ti 2p and Ni 2p peaks disappear, accompanied by a marked increase in C, N, and O content, confirming the successful formation of a PDA film on the NiTi surface. Upon introducing CuSO_4_ into dopamine solution, the Cu@PDA/NiTi spectra retain the C 1s, N 1s, and O 1s peaks, indicating the persistence of the PDA, and simultaneously display Cu 2p peaks. High-resolution Cu 2p spectra of 20Cu@PDA reveal strong Cu 2p_3/2_ and Cu 2p_1/2_ peaks characteristic of Cu^2+^, along with a weaker peak at 932.6 eV attributable to Cu0 (Fig. 1b).^37^ Consequently, the Cu@PDA films are composed of PDA, Cu^2+^, and Cu (0). Table S1 further demonstrates that the Cu content in the Cu@PDA films increase progressively with increasing *C*_CuSO_4__.

**Fig. 2:**
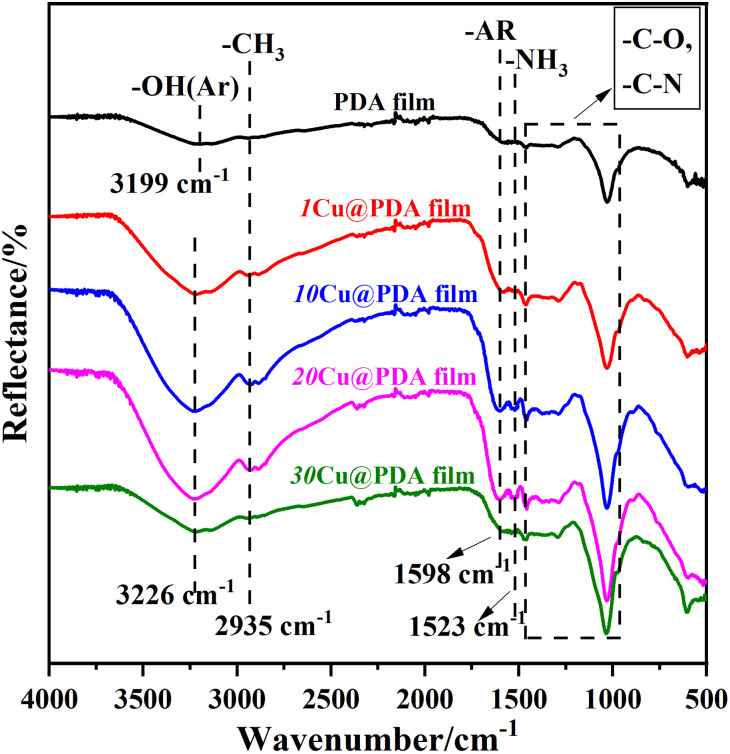
FTIR spectra of PDA film and Cu@PDA films constructed by adjusting *C*_CuSO_4__.

Fig. 2 presents the FTIR spectra of the PDA film and Cu@PDA films constructed by varying *C*_CuSO_4__. The PDA film exhibits distinct absorption peaks corresponding to the phenolic hydroxyl stretching vibrations of the catechol group (3199 cm^−1^), the stretching vibrations of aromatic rings (1598 cm^−1^) and C–H (2935 cm^−1^), as well as the N–H shearing vibrations of the amid group (1523 cm^−1^), moreover, a large relative absorbance in the 1500–1100 cm^−1^ region is attributed to C–O and C–N functional groups.^36,38^ In contrast, the peak at 3226 cm^−1^ in the Cu@PDA films showed a noticeable red shift compared with that in the PDA film, which could be attributed to a coordination interaction^39,40^ between Cu^2+^ and the functional groups in the PDA. Additionally, the absorption peaks of the Cu@PDA films closely resembled those of the PDA film, further confirming the presence of PDA within the Cu@PDA films.

**Fig. 3:**
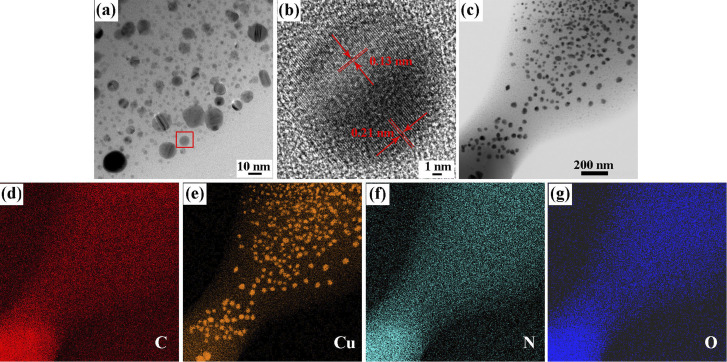
TEM and EDS-mapping images of 20Cu@PDA, (a) TEM image, (b) high-resolution TEM image and (c–g) EDS-mapping images.

The nanostructure of a representative coating (20Cu@PDA) was then examined by TEM. Fig. 3a shows the TEM image of 20Cu@PDA film, revealing dense, irregular nanoparticles with an average size of 10 nm. HRTEM image (Fig. 3b) displays lattice fringes of 0.13 nm and 0.21 nm, corresponding to the (220) and (111) planes of metallic Cu, confirming the presence of Cu nanoparticles.^41,42^ EDS mapping (Fig. 3c–g) further demonstrates the uniform distribution of C, N, O, and Cu throughout the film.

**Fig. 4:**
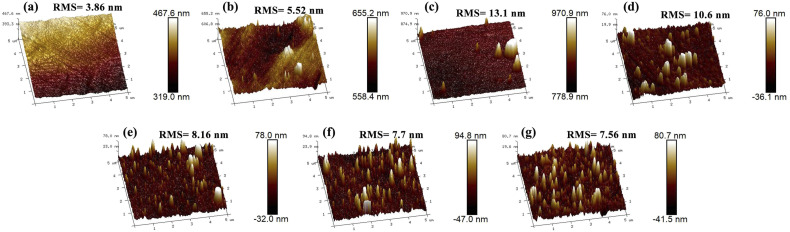
AFM images and RMS value of polished NiTi, PDA/NiTi and Cu@PDA/NiTi constructed by adjusting *C*_CuSO_4__, (a) polished NiTi, (b) PDA/NiTi, (c) 1Cu@PDA, (d) 5Cu@PDA, (e) 10Cu@PDA, (f) 20Cu@PDA and (g) 30Cu@PDA, respectively.

The surface topography of all samples was quantitatively characterized by AFM, with the images and the derived root-mean-square (RMS) roughness values are displayed in Fig. 4. The polished NiTi presents a smooth surface with an RMS roughness of 3.86 nm (Fig. 4a). The PDA/NiTi slightly increases roughness to 5.52 nm (Fig. 4b). By contrast, the incorporation of Cu species dramatically alters the surface morphology. For the Cu@PDA/NiTi, the RMS roughness first rise to a maximum of 13.1 nm for 1Cu@PDA (Fig. 4c) and then progressively decreases with increasing *C*_CuSO_4__, reaching 7.56 nm for 30Cu@PDA (Fig. 4g). This progressively evolution in surface roughness is a direct consequence of changing deposition dynamics governed by the Cu^2+^ concentration, as discussed in the formation mechanism (3.2 Formation mechanism). The observed nanoscale protrusions are a typical feature of metal-ion-incorporated PDA composite coatings, as reported in analogous systems.^37^

### 3.2 Formation mechanism

Surface modification using PDA film is initiated by dissolved oxygen in a mildly alkaline dopamine solution.^43,44^ In our previous study, Ag@PDA films were formed on NiTi alloy by immersing the alloy in to a dopamine–AgNO_3_ mixture.^34^ Here, an analogous bath containing dopamine and CuSO_4_ was employed to construct Cu@PDA films on NiTi alloy.

Although Cu^2+^ is a weaker oxidant than Ag^+^, it strongly chelates electron-donating groups. Upon CuSO_4_ addition, two parallel pathways operate: (I) Cu^2+^ ions, whose redox potential exceeds that of dissolved oxygen, are partially reduced to Cu (0) nanoparticles by dopamine *via* the reductive capacity of catechol groups, and (II) dopamine undergoes oxidative polymerization accelerated by Cu^2+^.^37^ As the reaction proceeding, the Cu^2+^ concentration decreases, the oxidizing capacity of the ions wanes and chelation becomes dominant. Continuous Cu^2+^ accelerated and oxygen-driven polymerization yields PDA oligomers that coordinate Cu^2+^ through catechol moieties (forming metal-phenolic networks, MPNs), which act as nucleation sites. This process leads to forming surface protrusions and increasing RMS roughness relative to pure PDA film, a morphology characteristic of metal-incorporated PDA coatings.^37^

**Fig. 5:**
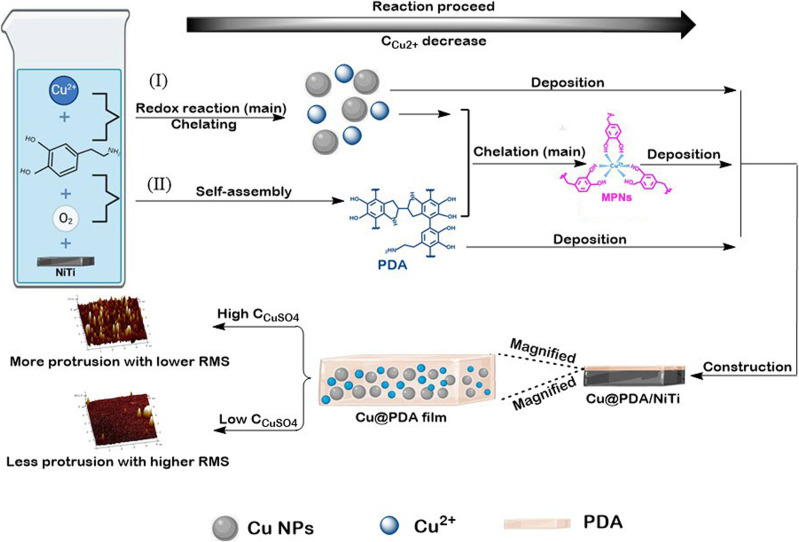
Schematic illustration of the formation mechanism of Cu@PDA/NiTi.

Meanwhile, dopamine consumed in Cu^2+^ reduction no longer contributes to film growth. Instead, residual dopamine, the generated Cu NPs, MPNs and dissolved oxygen jointly assemble the Cu@PDA film. Consequently, higher *C*_CuSO_4__ deplete more dopamine, reducing the amount of PDA available for film formation and RMS roughness, while simultaneously increasing protrusion density as observed. Fig. 5 illustrates schematic illustration of the formation mechanism of Cu@PDA/NiTi.

### 3.3 Corrosion resistance

**Fig. 6:**
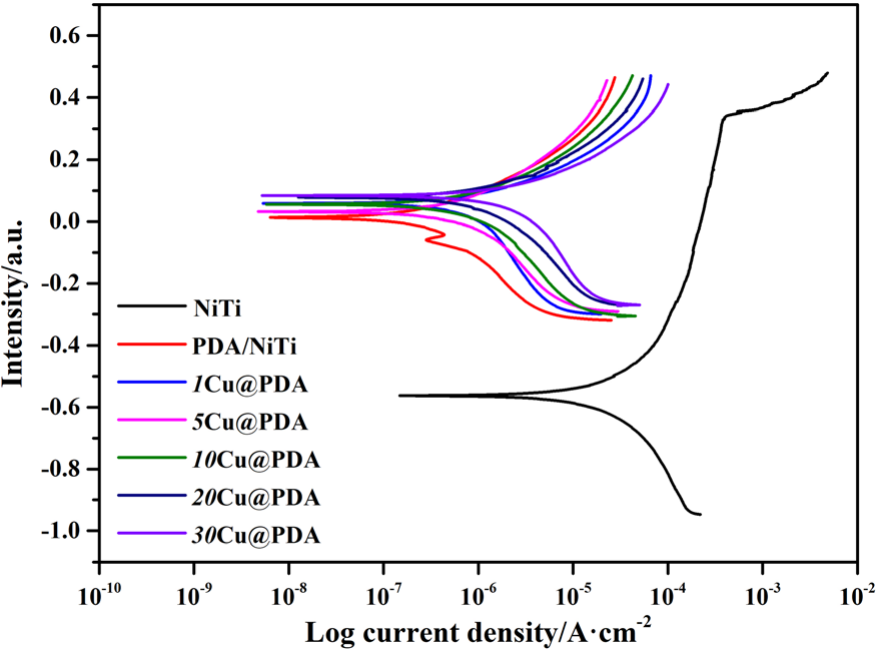
Polarization curves of polished NiTi, PDA/NiTi, and Cu@PDA/NiTi in Hank's solution.

Fig. 6 shows the potentiodynamic polarization curves of polished NiTi, PDA/NiTi and Cu@PDA/NiTi, and the corresponding electrochemical corrosion results are listed in Table S2. Polished NiTi exhibits the lowest corrosion resistance, with *E*_corr_ at −0.56 V, *I*_corr_ at 1.11 × 10^−5^ A cm^−2^, and *E*_pit_ at 0.33 V (Table S2). Among Cu@PDA/NiTi, higher *E*_corr_ and *I*_corr_ values are observed relative to PDA/NiTi, and these values rise modestly as *C*_CuSO_4__ increases from 1 to 30 mM. Nevertheless, all Cu@PDA/NiTi exhibits more positive *E*_corr_ and *E*_pit_ values, and lower *I*_corr_ than polished NiTi, indicating the Cu@PDA films can improve the corrosion resistance of the NiTi alloy.

Improving corrosion resistance is one of the important tasks in the surface modification of NiTi alloy.^45–47^ Numerous studies have confirmed that PDA films are able to improve substrate corrosion resistance.^32,33^ Here, a dopamine–CuSO_4_ bath was employed to construct Cu@PDA films on NiTi alloy. As *C*_CuSO_4__ rises, dopamine is progressively diverted to reduce Cu^2+^ to Cu NPs, decreasing the amount of PDA contributed to construct films, which elevates *I*_corr_. Simultaneously, the growing Cu NPs fraction lowers films density, further increasing *I*_corr_.

### 3.4 Contact angle measurements

The hydrophilic properties of implant materials are crucial as they significantly influence surface characteristics and bioactivity.^48–50^ Therefore, the contact angle method was employed to evaluate the hydrophilicity of Cu@PDA/NiTi. Fig. S1 shows the hydrophilicity of the polished NiTi, PDA/NiTi, and Cu@PDA/NiTi. Polished NiTi is relative hydrophobic, exhibiting a contact angle of 81.3° (Fig. S1a). After PDA deposition, the contact angle falls to 75.8° (Fig. S1b), indicating improved hydrophilicity. All Cu@PDA/NiTi are more hydrophilic than both polish NiTi and PDA/NiTi, and the contact angle declines from 71.1° (1Cu@PDA) to 56.2° (30Cu@PDA) as *C*_CuSO_4__ increases (Fig. S1c–g). This trend reflects the enhanced hydrophilicity conferred by higher *C*_CuSO_4__.

The PDA film is rich in phenolic hydroxyl and amino groups, promoting superior wettability^51,52^ relative to polished NiTi. Moreover, as shown in Section 3.1, elevated *C*_CuSO_4__ increases protrusion density, thereby raising the surface density of hydrophilic groups and further improving the hydrophilic properties.

### 3.5 *In vitro* antibacterial and cytocompatibility assessments

**Fig. 7:**
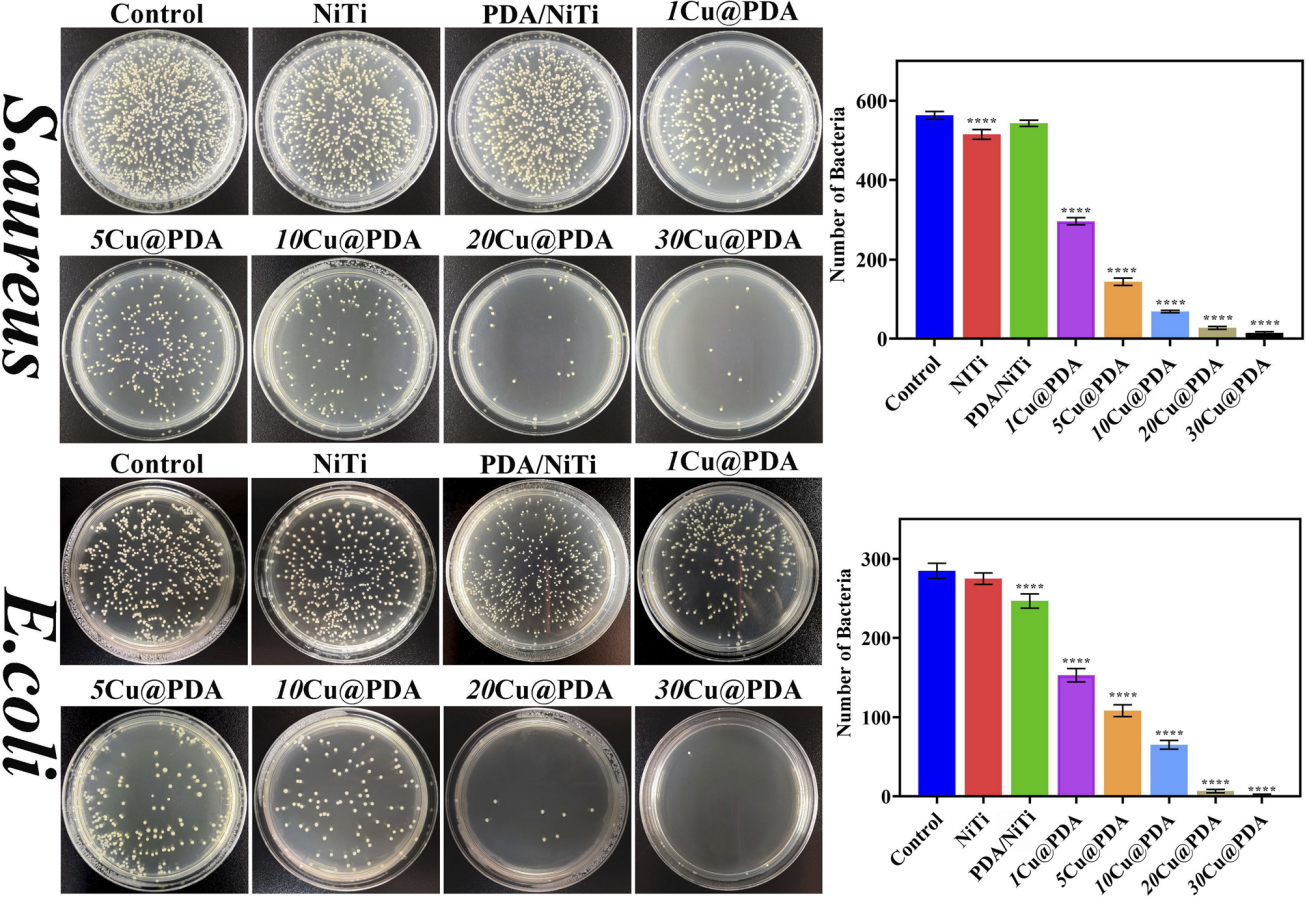
Representative colony forming images and analytical results of *S. aureus* and *E. coli* incubated with the samples at 37 °C for 24 hours. The results were obtained from the CFU counts (*n* = 3), where **** represents *P* < 0.0001 *vs.* control.

An effective antibacterial surface can mitigate post-surgical infections.^53,54^ To evaluate the antibacterial activity of Cu@PDA/NiTi constructed by adjusting *C*_CuSO_4__, *E. coli* and *S. aureus* were employed as test strains. Fig. 7 and Table S3 display the representative colony images, analytical results and AR for polished NiTi, PDA/NiTi and Cu@PDA/NiTi towards *S. aureus* and *E. coli*, respectively. Samples were incubated with bacterial suspensions (1 × 10^6^ CFU mL^−1^) at 37 °C for 24 hours. Overall, Cu@PDA/NiTi outperformed both polished NiTi and PDA/NiTi. As *C*_CuSO_4__ increased, antibacterial ratios rose from 46.3% to 99.4% against *E. coli* and from 47.5% to 97.3% against *S. aureus*, respectively (Table S3).

**Fig. 8:**
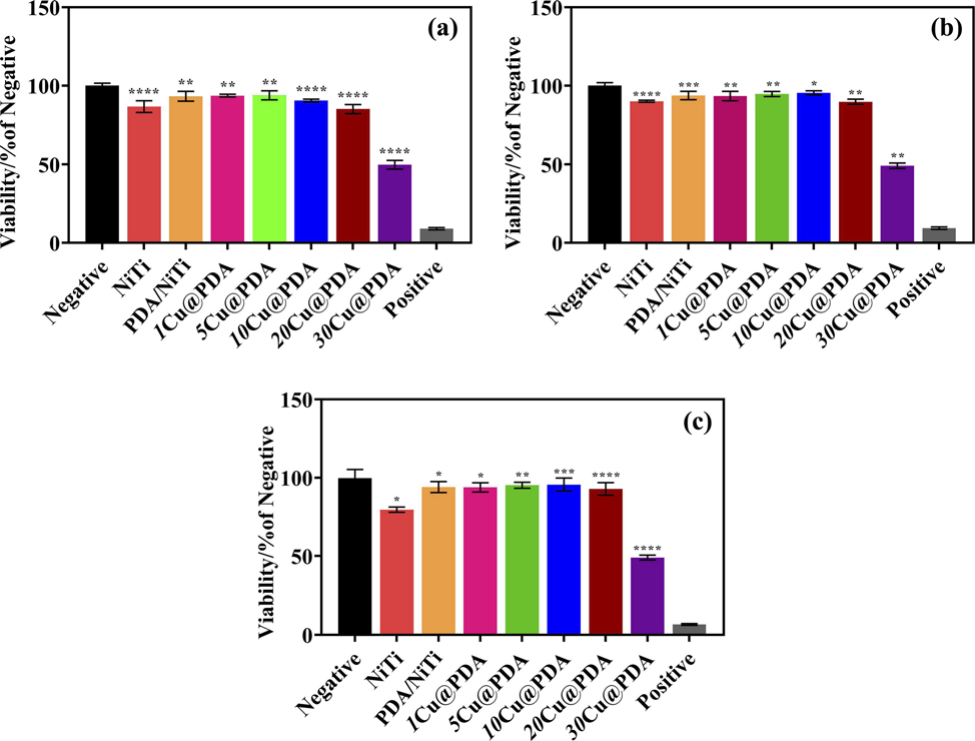
*In vitro* viability of NIH-3T3 incubated with samples for (a) 1 day, (b) 3 days and (c) 5 days (*n* = 3). Where * represents *P* < 0.05 *vs.* negative, ** represents *P* < 0.01 *vs.* negative, *** represents *P* < 0.001 *vs.* negative and **** represents *P* < 0.0001 *vs.* negative.

Biosafety is essential for clinical biomaterials.^55^ The *in vitro* cytocompatibility of polished NiTi, PDA/NiTi and Cu@PDA/NiTi was assessed against NIH-3T3 cells using the CCK-8 assay. As shown in Fig. 8, after 5 days of incubation, the polished NiTi exhibits a relativly low cell viability, amounting to only 75% of the negative control. Deposition of PDA film increased viability to above 90% of the negative control. In contrast, 1Cu@PDA, 5Cu@PDA, 10Cu@PDA and 20Cu@PDA all maintained viabilities above 90%, levels comparable to those of PDA/NiTi, demonstrating that the Cu@PDA/NiTi constructed by adjusting *C*_CuSO_4__ from 1 mM to 20 mM retain excellent cytocompatibility. In contrast, 30Cu@PDA exhibited viability below 50% after 5 days, indicating significant cytotoxicity.

**Fig. 9:**
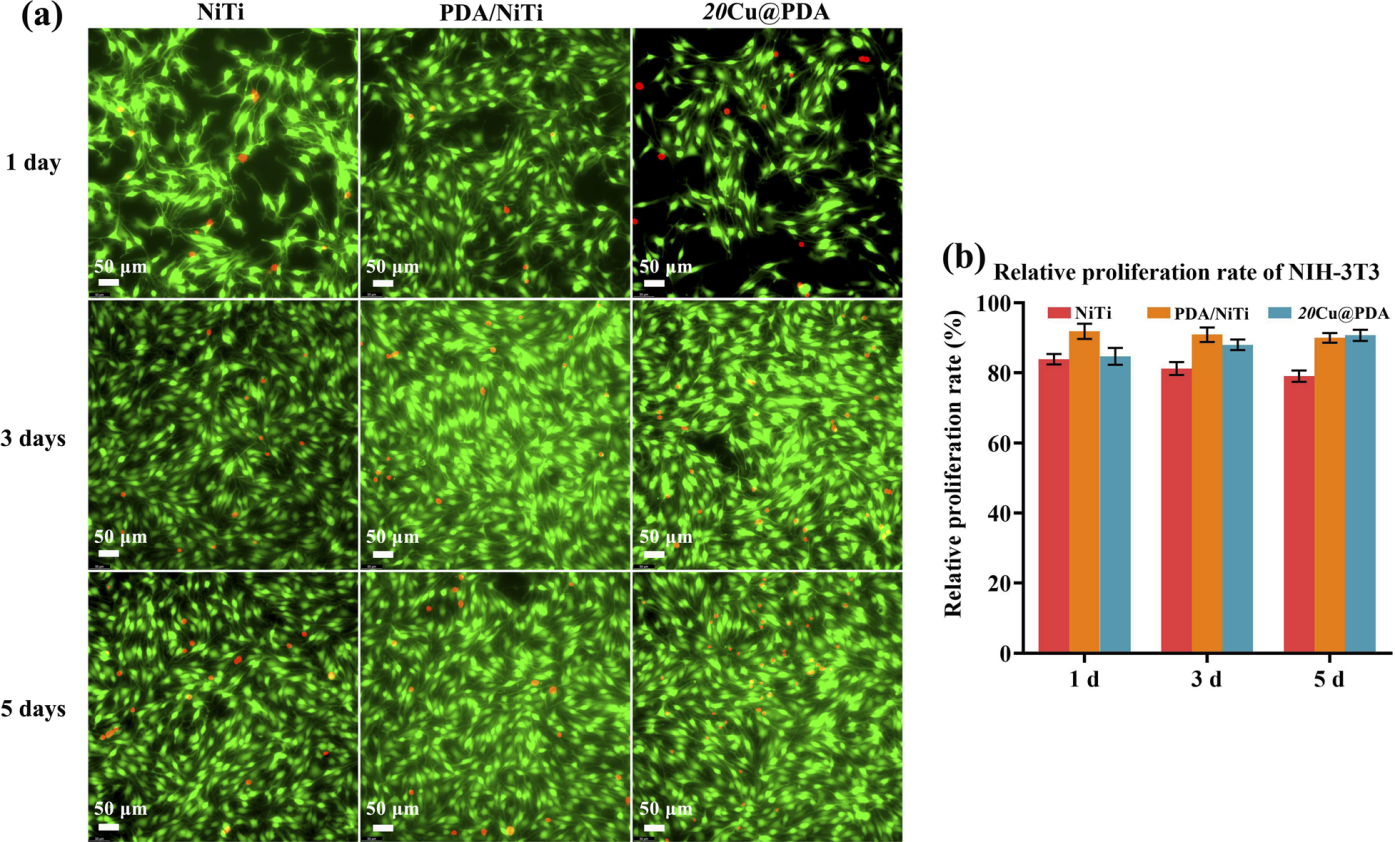
Fluorescence microscopy images (a) and relative proliferation rate (b) of NIH-3T3 cells incubated with polished NiTi, PDA/NiTi and 20Cu@PDA for 1 day, 3 days and 5 days.

Integrating corrosion, antibacterial and cytocompatibility results, the 20Cu@PDA displayed markedly enhanced corrosion resistance, excellent antibacterial activity and good cytocompatibility. Consequently, 20Cu@PDA was selected for live/dead staining with calcein-AM (live, green) and propidium iodide (dead, red) and compared with polished NiTi and PDA/NiTi. Fig. 9a reveals that live-cell fluorescence (green) increases progressively over 1, 3 and 5 days across all groups, yet the absolute cell count is higher on 20Cu@PDA and PDA/NiTi than on NiTi, indicating superior cell adhesion and proliferation. These observations align with the CCK-8 results.

### 3.6 Release behavior of Ni ions and Cu ions

**Fig. 10:**
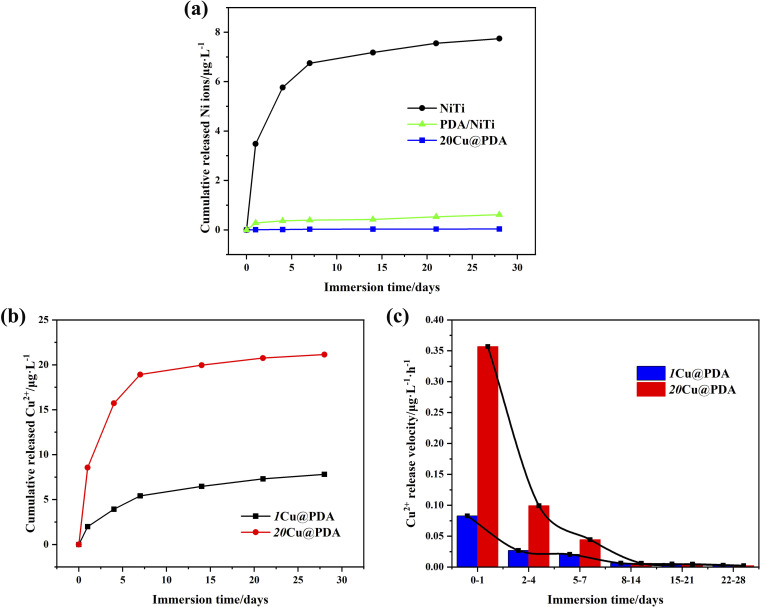
Ions release curves of the samples in PBS solution with prolonging the immersion time, (a) Ni ions release curves of polished NiTi, PDA/NiTi and 20Cu@PDA, (b) Cu^2+^ release curves of 1Cu@PDA AND 20Cu@PDA, and (c) Cu^2+^ release velocity of 1Cu@PDA and 20Cu@PDA.

Fig. 10a compares the Ni-ion release kinetics of polished NiTi, PDA/NiTi and 20Cu@PDA over 28 days of immersion. By contrast, the PDA film markedly suppresses the Ni-ion release, whereas 20Cu@PDA film provides an even stronger barrier, rendering cumulative Ni ions release virtually negligible relative to that of polished NiTi. This enhanced suppression is attributed to Cu^2+^ chelation, which reinforces cross-linking of PDA oligomers,^39,40,56^ thereby densifying the film and further blocking Ni ions release.

Fig. 10b and c present the release profile and release velocity of Cu^2+^ from 20Cu@PDA during the same 28 days of immersion. An initial fast release is observed within the first day, followed by a sharp decline over the next 7 days. This rapid early release is optimal for preventing or treating post-operative infection. Subsequently, the release rate decreases gradually for the remainder of the test, confirming the sustained antibacterial activity of 20Cu@PDA and its capacity to promote tissue healing.

Cu is an essential trace element in humans. Cu^2+^ can readily bind bacterial cell walls, disrupt membrane integrity and induce intracellular leakage, ultimately killing the bacteria. Cu^2+^ can also penetrate the cytoplasm, generate reactive oxygen species (ROS) and damage nucleic acids, proteins, and lipids, leading to bacterial death. Additionally, Cu nanoparticles serve as a sustained reservoir of Cu^2+^ ions and can also penetrate bacterial membranes, generating a synergistic antibacterial effect with the released ions.^26–29^

To translate these intrinsic antibacterial advantages into a durable surface function, we exploited a PDA matrix to immobilize both Cu^2+^ and Cu NPs while controlling Cu^2+^ release kinetics. Based on the combined evidence from XPS, FTIR and TEM, it is demonstrated that Cu^2+^ is chelated by PDA oligomers while being partially reduced to formed embedded Cu NPs. This process constructs a cross-linked Cu@PDA films on the NiTi alloy. Although the direct quantification of dopamine polymerization conversion in the presence of Cu^2+^ is methodologically challenging and seldom reported in similar system,^57,58^ the constructed films suppress Ni-ion release more effective because the additional Cu^2+^ cross-linking densifies the films compared with PDA film alone. Concurrently, this altered growth mechanism results in a characteristic nanostructured topography. The same architecture imposes a PDA-mediated diffusion barrier, thus, Cu^2+^ release is governed by PDA degradation kinetics, guaranteeing a prolonged antibacterial efficacy.^37,59^ With increasing *C*_CuSO_4__, the Cu content in the films increases, leading to a faster release rate during bacterial challenge and thereby higher antibacterial ratios. Meanwhile, elevated *C*_CuSO_4__ progressively oxidizes more dopamine, reducing the amount available for polymerization and weakening the inhibitory effect of PDA, which further accelerate Cu^2+^ release and thereby achieves tunable antibacterial activity through adjusting *C*_CuSO_4__.

### 3.7 *In vivo* biosafety evaluation

**Fig. 11:**
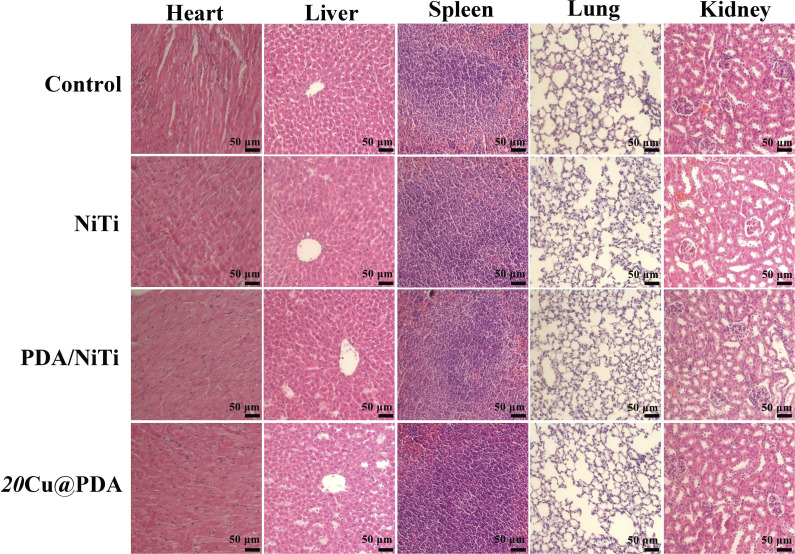
H&E staining of heart, liver spleen, lung, and kidney from control group, polished NiTi group, PDA/NiTi group and 20Ag@PDA group.

Long-term *in vivo* biosafety was assessed by subcutaneous implantation of polished NiTi, PDA/NiTi and 20Cu@PDA in mice for 28 days. As shown in Fig. 11, H&E staining of heart, liver, spleen, lung and kidney revealed no pathological alterations: hepatic lobules were intact and free of fatty degeneration or fibrosis; renal glomeruli exhibited normal architecture, myocardial fibers were orderly arranged, alveolar structures were clearly declined, and splenic white and red pulp were sharply defined with uniform lymphocyte distribution and no congestion or necrosis. Macroscopically, all organs exhibited typical morphology, devoid of swelling, hemorrhage, necrosis or abnormal proliferation (Fig. S2). These results collectively demonstrate that 20Cu@PDA is highly biocompatible and elicits no adverse systemic or local tissue responses.

### 3.8 Scalability and practical potential

The facile one-pot construction of Cu@PDA/NiTi is inherently suited for scaling. First, the single immersion step at room temperature is readily scalable and applicable to implants with complex shapes. Second, the tunable antibacterial activity can be achieved solely *via* adjusting initial concentration of CuSO_4_, enables the manufacturing of devices with tailored infection-fighting potency without altering the core process. In addition, coupled with the enhanced corrosion resistance, this facile strategy offers a practical and versatile avenue for producing safer NiTi implants.

## 4. Conclusion

In summary, we present a straightforward one-pot route to construct Cu@PDA/NiTi. By leveraging the simultaneous self-polymerization of dopamine and reduction of Cu^2+^, the method enables the tuning of antibacterial activity and confers an enhancement in the corrosion resistance of NiTi alloy, simply through adjustment of the initial concentration of CuSO_4_. The resulting Cu@PDA/NiTi deliver tunable bacterial efficacy against *E. coli* and *S. aureus*, virtually eliminate Ni-ion release, and retain full cytocompatibility. This work provides a viable path to overcoming the long-standing challenge of balancing biosafety and antimicrobial efficacy in NiTi implants, offering a facile, readily scalable strategy for customizing implants for diverse clinical scenarios, and providing a blueprint for next-generation metallic biomaterials.

## Author contributions

Investigation, methodology and formal analysis: Tong Liu, Hao Liu, Leheng Ren and Haojie Sun. Resource, funding and conceptualization: Yongkui Yin and Ying Li. Data curation and validation: Tong Liu. Project administration: Hongfeng Liu and Ying Li. Writing-original draft and Writing-review & editing: Yongkui Yin and Ying Li. All authors have read and agreed to the published version of the manuscript.

## Conflicts of interest

There are no conflicts to declare.

## Supplementary Material

RA-016-D5RA08002A-s001

## Data Availability

Data that support the findings of this study are available from the corresponding author upon request. Supplementary information: surface element contents, electrochemical corrosion test results, antibacterial ratios, water contact angles of the samples, and representative histological photographs tissues. See DOI: https://doi.org/10.1039/d5ra08002a.

## References

[cit1] Patel A., Pavlou G., Mujica-Mota R. E., Toms A. D. (2015). Bone Joint J..

[cit2] Alontseva D., Azamatov B., Safarova Y., Voinarovych S., Nazenova G. (2023). Coatings.

[cit3] Mazur F., Zhou Y., Ng G., Fan Q., Pham A.-H., Boyer C., Chandrawati R. (2024). Commun. Mater..

[cit4] Assad M., Lombardi S., Berneche S., Desrosiers E. A., Yahia L. H., Rivard C. H. (1994). Ann. Chir..

[cit5] Jalali M., Mohammadi K., Movahhedy M. R. R., Karimi F., Sadrnezhaad S. K., Chernyshikhin S. V. V., Shishkovsky I. V. V. (2023). Met. Mater. Int..

[cit6] Nespoli A., Passaretti F., Szentmiklósi L., Maróti B., Placidi E., Cassetta M., Yada R. Y., Farrar D. H., Tian K. V. (2022). Metals.

[cit7] Auricchio F., Petrini L., Pietrabissa R., Sacco E. (2003). Comput. Model. Eng. Sci..

[cit8] Ozyurek H., Elbay M., Ozyurek T. (2023). Front. Mater..

[cit9] Jamshidi P., Panwisawas C., Langi E., Cox S. C., Feng J., Zhao L., Attallah M. M. (2022). J. Alloys Compd..

[cit10] Du T., Liu J., Dong J., Xie H., Wang X., Yang X., Yang Y. (2024). Front. Bioeng. Biotechnol..

[cit11] Hua W., Fu G., Guan W., Wang Q., Zhu B., Meng Z. (2025). Mater. Today Commun..

[cit12] Liang P., Li P., Yang Y., Yang K., Mao C., Chi H., Zhang J., Yu Z., Xu Z., Guo Y., Ren L. (2024). Ceram. Int..

[cit13] Yang Y., Xu Z., Sha P., Li P., Xu Z., Guo Y., Yu Z., Zhang Z., Ren L. (2025). Adv. Compos. Hybrid Mater..

[cit14] Li X., Yang Y., Shen H., Zhou M., Huang B., Cui L., Hao S. (2025). Colloids Surf., B.

[cit15] Nazarov D. V., Kozlova L. A., Yudintceva N. M., Ovcharenko E. A., Rudakova A. V., Kirichenko S. O., Rogacheva E. V., Kraeva L. A., Borisov E. V., Popovich A. A., Maximov M. Y. (2024). Appl. Surf. Sci..

[cit16] Gnanasekar S., He X., Nagay B. E., Xu K., Rao X., Duan S., Murugesan S., Barao V. A. R., Kang E.-T., Xu L. (2025). Bioact. Mater..

[cit17] Zhao C., Liu W., Zhu M., Wu C., Zhu Y. (2022). Bioact. Mater..

[cit18] Bedouet L., Beilvert A., Servais E., Pascale F., Ghegediban S. H., Namur J., Moine L. (2025). ACS Infect. Dis..

[cit19] Javed S., Barkatullah Z., Yaqoob E. (2024). Neurosurg. Rev..

[cit20] Alshammari Y., Elkork N., Moussa L., Esmaeil F., Saeed M., Alsarraf M., Alfarhan A., Alrashidi M. A., Bolzoni L. (2025). Crit. Rev. Solid State Mater. Sci..

[cit21] Ortega-Nieto C., Losada-Garcia N., Prodan D., Furtos G., Palomo J. M. (2023). Nanomaterials.

[cit22] Li X. J., Shang H. L., Xiong Y., Luan Y., Wang D., Du X. (2025). Chem. Eng. J..

[cit23] Cao H., Zhang W., Meng F., Guo J., Wang D., Qian S., Jiang X., Liu X., Chu P. K. (2017). ACS Appl. Mater. Interfaces.

[cit24] Mitra S., Das A., Sen S., Mahanty B. (2018). World J. Microbiol. Biotechnol..

[cit25] Gao J., Xu J., Wen S., Hu J., Liu H. (2015). Microporous Mesoporous Mater..

[cit26] Duan X., Li G., Jia Z., Xie H., Li X. (2024). J. Mol. Struct..

[cit27] Wang F., Wang X., Li S., Yang Q., Mu H., Li J., Yang Y. (2025). Carbohydr. Polym..

[cit28] Bondarenko O., Ivask A., Kaekinen A., Kahru A. (2012). Environ. Pollut..

[cit29] El-Sherbiny G. M., Shehata M. E., Kalaba M. H. (2025). Biotechnol. Rep..

[cit30] Islam M. S., Akter N., Rahman M. M., Shi C., Islam M. T., Zeng H. B., Azam M. S. (2018). ACS Sustain. Chem. Eng..

[cit31] Shi L., Santhanakrishnan S., Cheah Y. S., Li M., Chai C. L. L., Neoh K. G. (2016). ACS Appl. Mater. Interfaces.

[cit32] Hong M. S., Park Y., Kim T., Kim K., Kim J. G. (2020). J. Materiomics.

[cit33] Ding Z. H., Fatollahi-Fard F., Kwon I. S., Pistorius P. C., Bettinger C. J. (2018). Adv. Eng. Mater..

[cit34] Yin Y., Li Y., Cai W., Sui J. (2019). RSC Adv..

[cit35] Ahmed R. A., Fadl-allah S. A., El-Bagoury N., El-Rab S. M. F. G. (2014). Appl. Surf. Sci..

[cit36] Jia Z., Xiu P., Li M., Xu X., Shi Y., Cheng Y., Wei S., Zheng Y., Xi T., Cai H., Liu Z. (2016). Biomaterials.

[cit37] Meng L., Huang C., Liu X., Qu H., Wang Q. (2023). Front. Bioeng. Biotechnol..

[cit38] Yu F., Chen S., Chen Y., Li H., Yang L., Chen Y., Yin Y. (2010). J. Mol. Struct..

[cit39] Wang Y. A., Liu T., Zhong G. Q. (2019). Carbohydr. Polym..

[cit40] Khalil M. M., El-Sayed A. H., Masoud M. S., Mahmoud E. M., Hamad M. A. (2021). J. Mater. Res. Technol..

[cit41] Li X. J., Hao C. C., Lei Q. Q. (2012). Mater. Res. Bull..

[cit42] Hirai M., Kumar A. (2007). J. Electron. Mater..

[cit43] Lee H., Dellatore S. M., Miller W. M., Messersmith P. B. (2007). Science.

[cit44] Zhang C., Lv Y., Qin W. Z., He A., Xu Z. K. (2017). ACS Appl. Mater. Interfaces.

[cit45] Zhu J. N., Li Z. Y., Rahimi E., Yan Z. R., Ding Z. Y., Mol A., Popovich V. (2025). Corros. Sci..

[cit46] Tuissi A., Rondelli G., Bassani P. (2015). Shap. Mem. Superelasticity.

[cit47] Bayat N., Sanjabi S., Barber Z. H. (2011). Appl. Surf. Sci..

[cit48] Wang S., Zhang M., Liu L. L., Xu R. W., Huang Z. L., Shi Z. A., Liu J. C., Li Z., Li X. H., Hao P., Hao Y. Q. (2022). Front. Bioeng. Biotechnol..

[cit49] Li S., Jin Y. Y., Bai S. X., Yang J. (2022). Materials.

[cit50] Bhardwaj T., Shukla M., Prasad N. K., Paul C. P., Bindra K. S. (2020). Met. Mater. Int..

[cit51] Zhang Y. X., Wang D., Xu Y., Wen L., Dong J., Wang L. M. (2024). Coatings.

[cit52] Zhang H., Hu H. Y., Ye W. C., Zhou F. (2011). J. Appl. Polym. Sci..

[cit53] Hadem H., Ojha A. K., Mukherjee S., Prasad P. S., Biswas A., Dhara S., Das S., Das K. (2025). Colloids Surf., A.

[cit54] Sörensen J. H., Lilja M., Sörensen T. C., Åstrand M., Procter P., Fuchs S., Stromme M., Steckel H. (2014). J. Biomed. Mater. Res., Part B.

[cit55] Saito N., Haniu H., Aoki K., Nishimura N., Uemura T. (2022). Adv. Sci..

[cit56] Yang Z. L., Yang Y., Xiong K. Q., Wang J., Lee H., Huang N. (2018). Chem. Mater..

[cit57] Ball V., Gracio J., Vila M., Singh M. K., Metz-Boutigue M. H., Michel M., Bour J., Toniazzo V., Ruch D., Buehler M. J. (2013). Langmuir.

[cit58] Zhou X. M., Gao S. J., Huang D., Lu Z. B., Guan Y., Zou L., Hu K. L., Zhao Z., Zhang Y. J. (2022). Macromol. Rapid Commun..

[cit59] Liu G. Y., Li K. J., Wang H. B., Ma L., Yu L., Nie Y. (2020). ACS Appl. Mater. Interfaces.

