# Supplementary material for: Facile one-pot construction of copper-loaded polydopamine films on NiTi alloy for adjustable antibacterial activity and enhanced corrosion resistance
Source: RSC Adv. 2026 Jan 21;16(5):4555–66. doi: 10.1039/d5ra08002a (PMC12821150; doi:10.1039/d5ra08002a)
Supplement: RA-016-D5RA08002A-s001 [file RA-016-D5RA08002A-s001.pdf]

Supplementary Information (SI) for RSC Advances.
This journal is © The Royal Society of Chemistry 2026

Supplementary Information for:

Facile One-Pot Construction of Copper-Loaded Polydopamine Films on NiTi Alloy for Adjustable Antibacterial Activity and Enhanced Corrosion Resistance

Ying Li [a,c], Hongfeng Liu [b], Tong Liu [a], Hao Liu [a], Leheng Ren [a], Haojie Sun [a] and Yongkui Yin [a,*]

[a]College of Life Science, Mudanjiang Medical University, Mudanjiang, 157011, Heilongjiang Province, PR China

[b]*School of Basic Medicine, Mudanjiang Medical* University, Mudanjiang 157011, Heilongjiang Province, PR China

[c]School of Health Management, Mudanjiang Medical University, Mudanjiang, 157011, Heilongjiang Province, PR China

*Corresponding author

Email address: yinyongkui@mdjmu.edu.cn

Table S1 Surface element contents of polished NiTi, PDA/NiTi and Cu@PDA/NiTi constructed by adjusting C_{CuSO4}.

Samples	C (at.%)	O (at.%)	N (at.%)	Cu (at.%)	Ni (at.%)	Ti (at.%)
NiTi	28.39	40.83	3.7	-	12.23	14.81
PDA/NiTi	44.41	49.74	6.12	-	-	-
*1*Cu@PDA	41.43	46.77	7.59	-	-	-
*5*Cu@PDA	40.77	41.82	6.11	11.3	-	-
*10*Cu@PDA	35.99	36.82	5.67	21.52	-	-
*20*Cu@PDA	33.14	37.45	6.25	23.16	-	-
*30*Cu@PDA	30.39	36.05	6.85	26.71	-	-

Table S2 Electrochemical corrosion results of polished NiTi, PDA/NiTi and Cu@PDA/NiTi in Hank's solution.

Samples	I_{corr} (A/cm^{-2})	E_{corr} (V vs. SCE)	E_{pit} (V vs. SCE)
NiTi	1.11×10^{-5}	-0.56	0.33
PDA/NiTi	$(1.74\pm0.30)\times10^{-7}$	0.01±0.03	-
*1*Cu@PDA	$(2.43\pm0.28)\times10^{-7}$	0.05±0.03	-
*5*Cu@PDA	$(2.57\pm0.31)\times10^{-7}$	0.03±0.03	-
*10*Cu@PDA	$(2.59\pm0.36)\times10^{-7}$	0.05±0.03	-
*20*Cu@PDA	$(2.47\pm0.37)\times10^{-7}$	0.07±0.04	-
*30*Cu@PDA	$(3.24\pm0.39)\times10^{-7}$	0.08±0.04	-

Table S3 The AR of the samples towards *S.aureus* and *E.coli.*

Samples	*S.aureus* (AR)	*E. coli.* (AR)
NiTi	8.53±2.12%	3.53±2.21%
PDA/NiTi	3.55±1.21%	13.47±3.15%
*1*Cu@PDA	47.47±1.55%	46.32±4.92%
*5*Cu@PDA	74.43±1.70%	62.01±2.62%
*10*Cu@PDA	87.80±0.53%	77.09±1.94%
*20*Cu@PDA	95.15±1.21%	97.66±0.73%
*30*Cu@PDA	97.34±1.55%	99.41±0.41%

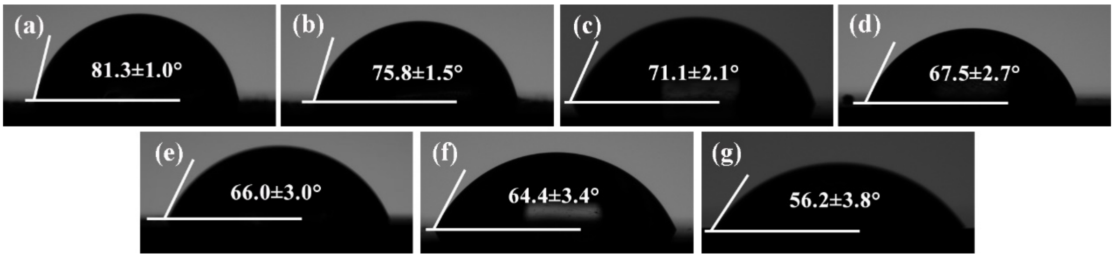

Fig. S1 Contact angles of polished NiTi, PDA/NiTi, and Cu@PDA/NiTi constructed by adjusting CCuSO4, (a) polished NiTi, (b) PDA/NiTi, (c) *1*Cu@PDA, (d) *5*Cu@PDA, (e) *10*Cu@PDA, (f) *20*Cu@PDA and *30*Cu@PDA, respectively.

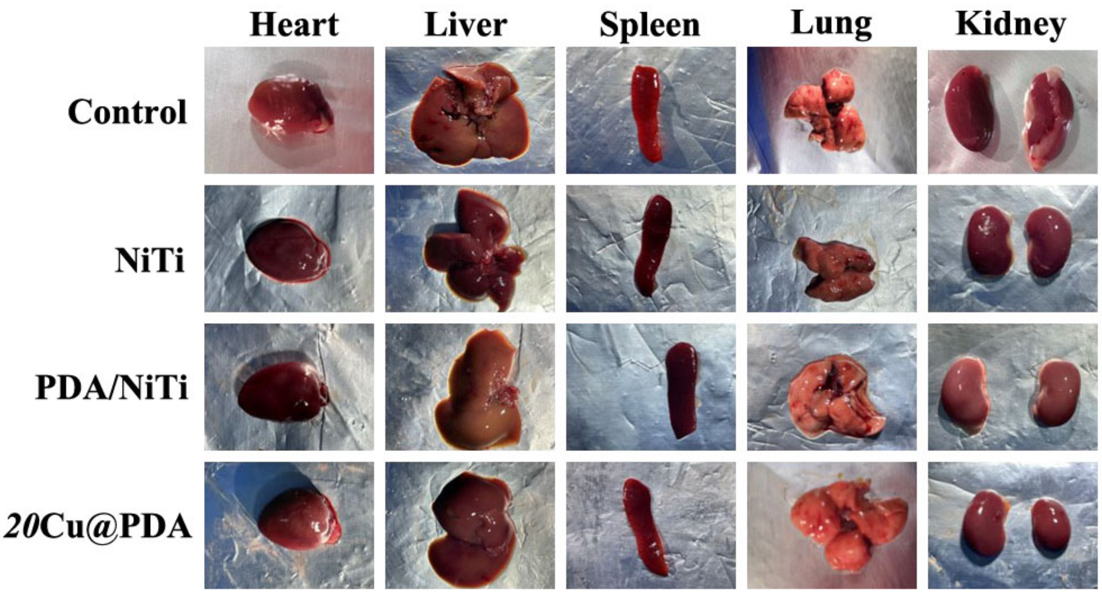

Fig. S2 Representative photographs of heart, liver spleen, lung, and kidney from control, polished NiTi, PDA/NiTi and *20*Cu@PDA groups.
